# Pediatric Gastroenterology Research

**DOI:** 10.3390/life13091810

**Published:** 2023-08-25

**Authors:** Vasile Valeriu Lupu, Ömer Faruk Beşer, Simona Gurzu, Gabriela Stefanescu, Iuliana Magdalena Starcea, Anca Adam Raileanu, Alina Popp, Ancuta Lupu

**Affiliations:** 1 Department of Pediatrics, “Grigore T. Popa” University of Medicine and Pharmacy, 700115 Iasi, Romania; 2 Department of Pediatric Gastroenterology, Hepatology, and Nutrition, Cerrahpasa Medical Faculty, Istanbul University Cerrahpasa, Istanbul 34776, Turkey; 3 Department of Pathology, George Emil Palade University of Medicine, Pharmacy, Science and Technology, 540012 Targu Mures, Romania; 4 Department of Gastroenterology, “Grigore T. Popa” University of Medicine and Pharmacy, 700115 Iasi, Romania; 5 Department of Pediatrics, “Carol Davila” University of Medicine and Pharmacy, 020021 Bucharest, Romania

For several decades, before the 19th century, pediatric pathology was considered to be an annex of adult pathology and treated as a secondary matter in medical practice. Knowledge accumulated over an extended time revealed that children’s pathology is fundamentally different from that found in adults’ in terms of gravity, evolution, severity degrees, disease frequency, capacity of recovery and long-term outcomes, including the influence exerted on the human organism’s capacity for growth and development [1].

It is obvious that children are not considered miniature adults as they have their own particular type of response to any kind of pathological aggression. As a result of these new acquired findings, in the second half of the 19th century, pediatrics separated from other medical specialties, as a specialty of its own. The extraordinary advances in recently improved science (physics, chemistry, biology) and medical technology, new technical means of medical imaging and laboratory work, the etiopathogenic, morphopathologic and physyiopathologic comprehension, have all broadened the sphere of knowledge in pediatrics and demanded the introduction of new pediatric subspecialities that almost match all adult compartments in diversity [2].

It is a favorable context for the onset and development of pediatric gastroenterology together with subspecialities, such as cardiology, pulmonology, nephrology, neurology and others. Each one of these subspecialities has its own characteristics but they all influence the growth and development of children. Although a relatively new specialty, pediatric gastroenterology claims its status starting with the 1990s of the last century, simultaneous with the decline in acute infectious pathology (bacterial, parasitic, viral) and its associated dehydration that caused so much pressure with acid–base and hydroelectrolytic rebalancing [3,4].

Behind these acute disorders is the existence of chronic pathology, which was often hidden in its entirety. This pathology was highly prevalent in daily practice and demanded to be recognized, classified and treated as an individual entity. The progression in endoscopic exploration capacity and imaging in general, as well as real-time video investigation, will only expand, especially in the digestive pathology field [5,6]. This phase was quite predictable, as the gastrointestinal tract represents the largest contact surface with the external environment (almost 400 square meters); otherwise, it is exposed to various types of aggression (physical, chemical, biological). Furthermore, the complex nature of the physiological, etiopathogenic and physiopathological processes required a histoenzymatic perspective that, today, can be applied when exploring every section of the digestive system [7].

Consequently, the digestive pathology approach can no longer be conceived without an endoscopy specialist’s help in describing the lesion, in the absence of the pathologist’s appreciation of the morphological alteration and without the gastroenterologist who gathers together the whole data. The mission of the latter is the most important as they are the one to associate the clinical symptoms with the morphopathological and physiopahological transformation, to make the diagnosis and, of course, to choose the appropriate therapy. Through this collaboration, significant advances have been made in the gastroenterology field. The underlying mechanisms of many digestive disorders and, specifically, numerous nosocomial entities have been described, such as gastroesophageal reflux (GER) and the associated reflux pathology, gastritis and the gastroduodenal ulcer, malabsorption syndromes, gut chronic inflammatory disorders, alimentary allergies and intolerances and, additionally, the digestive manifestations generated by other organs or system distress. Whether or not this pathology is associated or whether its manifestation is simultaneous, the appropriate assessment can only be made by a gastroenterology specialist, who can offer the right therapeutic option [1].

In-depth knowledge on pathogenic mechanisms has facilitated research in the therapeutic department as well. Novelties in this area, such as proton pump inhibitors, have been successful against several diseases, including gastroduodenal ulcers, from the surgery department (to whom the gastroduodenal ulcer was invariably addressed to) to a medical option of treatment, noninvasive and non-mutilating. Moreover, investigation options have individualized a series of disorders that can benefit from diagnostic and therapeutic guidelines, otherwise well founded by their results [8].

As already mentioned, during the last half of the century, the field of gastroenterology was dominated by two major achievements: the adaptation of fiberoptics to gastrointestinal endoscopy followed by the discovery of *Helicobacter pylori* (*H. pylori*) in 1982 [9]. Since its official introduction into medical practice, until today, there has been continuous research on understanding the pathogenetic mechanisms of *H. pylori*, and the therapeutic approach is continuously updated, as *H. pylori* infection remains one of the most appealing subjects in gastroenterology. Usually, *H. pylori* infection is acquired during childhood and persists as chronic gastritis if the bacterial agent is not eradicated. Recent epidemiological data on *H. pylori* among the pediatric population have shown that there has been a decrease in the prevalence of *H. pylori* infection, possibly due to environmental changes and current nutrition; however, the global prevalence remains high, signaling the importance of the condition [10,11,12]. Although for *H. pylori* infection diagnosis, there are several non-invasive tests available, invasive tests or endoscopic techniques along with an appropriate specimen increase the accuracy of the results [13,14]. Moreover, these invasive methods offer a better understanding of the physiopathological mechanisms that stand behind *H. pylori* infection and its association with several disorders.

It is well known that *H. pylori* infection represents a risk factor for other gastrointestinal conditions, such as gastroesophageal reflux disease, duodenal ulcers and gastric malignancy. Extra-digestive entities have also been correlated with *H. pylori* infection, including diseases from the autoimmune spectrum, cardiovascular diseases, pancreatic and colonic diseases, hepato-biliary system diseases, bronchiectasis, diabetes mellitus, neurological diseases and hematological diseases, such as iron-deficiency anemia [11,15,16,17,18,19]. Several mechanisms, like chronic inflammation, gastrointestinal microbiota alteration and its interplay between the metabolic, immune and neuroendocrine system, have been mentioned in order to explain these associations [20,21,22]. However, the intricate role of *H. pylori* infection in health and disease remains a subject of further research in gastroenterology, as current results are contradictory.

As already mentioned above, GER- and reflux-associated conditions represent another category of disorders that benefited from the introduction of the endoscopic technique in gastroenterology and currently dominate upper gastrointestinal tract pathology. The correlation between pH-metry and endoscopic lesions is extremely useful in children with atypical gastroesophageal reflux symptoms [23,24,25]. GER has been associated with respiratory symptoms, such as chronic cough, obstructive apnea episodes, recurrent otitis media and recurrent respiratory tract infections [26,27]. Furthermore, it has been recognized as a triggering factor in asthma [28,29], and evidence describes GER as one of the causes of recurrent wheezing in non-asthmatic children as well [30,31]. Dental caries, dry mouth, burning sensation, halitosis, mucosal erythema of the palate and uvula are typical oral symptoms in GERD and must be taken into consideration when classic symptoms of GER are absent [32]. Other GER symptoms, as described in the literature, might appear as sleep disturbances, agitation and crying episodes, neck hyperextension, arching and rigidity or irritability [33,34]. Nutritional consequences, such as growth impairment and iron deficiency anemia, can also be considered signs of GER, proving its complex nature [35,36].

Celiac disease (CD) is another entity that has benefited from the development of pediatric gastroenterology and the advances of medical technology. From a diagnosis that relied on the association of clinical symptomatology and the consumption of alimentary gluten, to histologic anomalies, serum antibodies and special HLA haplotypes, CD perfectly illustrates the need for a periodic update of medical guidelines [37]. Once considered to be a rare disease, CD is now considered a common disorder in pediatric patients with proof of increasing incidence. In recent decades, a various clinical pattern with widespread manifestation of the disease outside the gut has been observed. The early recognition of these features in children with CD and scarce gastrointestinal symptoms is still a challenge for the practitioner, despite the progress made in understanding the disease’s underlying mechanisms [37,38,39,40]. CD represents a chronic inflammatory disease of the small bowel mediated by immune responses to triggering peptides from dietary gluten, and it appears in genetically susceptible individuals. Both genetic predisposition and gluten exposure are necessary factors in CD pathogenesis, but their presence is not sufficient for disease development. Current evidence reveals that several additional environmental factors displayed their potential role as co-factors in CD progression through their ability to modulate the intestinal microbiome composition. In line with the purpose of individualized medical management of the patient, there is a particular focus on microbiota manipulation as both preventative and therapeutic options [38,39,40].

Another gastroenterological entity, inflammatory bowel disease (IBD), stands for a spectrum of disorders, including Crohn’s disease (CD) and ulcerative colitis (UC). With an incidence rise across the globe, particularly in children, there is an urgent need to improve the prognosis and quality of life of affected children. Pediatric patients come with many particular challenges, including growth impairment, pubertal delay, the psychology of adolescence and development of body image, adding more pressure on the healthcare provider [41]. While the precise disease characterization represents the basis of the modern treatment of IBD, endoscopy is the mainstay of disease assessment and colorectal cancer surveillance, suggesting a major role for medical technology in the management of IBD. This particular aspect of disease diagnosis and surveillance might come as a challenge in settings with a lack of resources and infrastructure. As for IBD treatment, endoscopic or biochemical remission, rather than clinical remission, represents the main therapeutic goal because, frequently, intestinal inflammation persists despite the resolution of abdominal symptomatology. In light of these issues specific to pediatric practice, there is a call for research advances such as large-scale pediatric trials, necessary in order to introduce new therapies to pediatric patients as well as new scientific knowledge meant to upgrade IBD endoscopy and histology, improving disease characterization and patient care [42].

The introduction of endoscopy in gastroenterology and the recognition of the pathogenic role of *H. pylori*, nearly 50 years ago, has changed the medical perspective on the upper gastrointestinal tract pathology. But the field of pediatric gastroenterology is evolving rapidly, and the continued progress of technology reveals new aspects of different disease’s underlying mechanisms. Transforming the optimal clinical management pathways, in different settings, is a matter of debate and a subject for future research.
